# Fluorescent proteins misreport ribosome abundance under translation inhibition

**DOI:** 10.64898/2026.09.17.752513

**Published:** 2026-09-22

**Authors:** Aniket Zodage, Justin Chen, Nabigha Mogharbel, Hidde de Jong, Suckjoon Jun

## Abstract

Fluorescent proteins are widely used as quantitative reporters of protein abundance in living cells. Here we show that this relationship can break down under translation inhibition: the cellular ribosomal abundance can increase while fluorescence intensity remains unchanged or even decreases. Using fluorescent reporters of ribosome abundance in *Escherichia coli* and *Bacillus subtilis* , we find that cellular fluorescence intensity quantitatively tracks ribosome abundance, inferred by RNA-to-protein ratio, under nutrient-limited growth but that is no longer true during chloramphenicol treatment. In *E. coli* , the discrepancy occurs with transcriptional and translational reporters and with both ribosomal protein and ribosomal RNA promoters. To distinguish changes in fluorescence output per reporter from changes in reporter abundance, we constructed a fluorescent protein–LacZ dual reporter in which fluorescence and an independent enzymatic estimate of reporter abundance are obtained from the same protein. Under translation inhibition, LacZ-derived reporter abundance increases whereas fluorescence intensity remains approximately constant, showing that fluorescence output per unit reporter abundance decreases. In a companion study, Bakshi and colleagues demonstrate that the effect extends to other fluorescent proteins and other translation inhibitors, indicating that it is not specific to a particular fluorescent protein or translation inhibitor. Our results show that fluorescent-protein calibration can be condition-dependent and cannot be assumed to transfer across physiological perturbations.

## Full Text Availability

The license terms selected by the author(s) for this preprint version do not permit archiving in PMC. The full text is available from the preprint server.

