# Supplementary material for: Fluorescent proteins misreport ribosome abundance under translation inhibition
Source: bioRxiv. 2026 Oct 4:2026.09.17.752513. Originally published 2026 Sep 22. Preprint. [Version 2] doi: 10.64898/2026.09.17.752513 (PMC13622061; doi:10.64898/2026.09.17.752513)
Supplement: Supplementary file 1 [file NIHPP2026.09.17.752513v2-supplement-1.pdf]

bioRxiv preprint doi: https://doi.org/10.64898/2026.09.17.752513; this version posted October 5, 2026. The copyright holder for this preprint (which was not certified by peer review) is the author/funder, who has granted bioRxiv a license to display the preprint in perpetuity. It is made available under a CC-BY 4.0 International license.

Supplementary Material

SI note 1 - Fluorescence intensity and LacZ activity from the reporters are expected to be proportional to the underlying ribosomal abundance (RNA-to-protein mass ratio)

Definitions

In the introduction section, we defined ribosomal abundance as the total ribosomal protein mass (M_{rib}) as a fraction of total protein mass ($M_{protein}$) in a cell:

$$\text{Ribosomal abundance} \equiv \frac{M_{\text{rib}}}{M_{\text{protein}}}.$$

We now define the RNA-to-protein ratio (ρ) and fluorescence intensity (f) of a cell as follows :

$$\rho \equiv \frac{M_{\text{RNA}}}{M_{\text{protein}}},$$
$$f \equiv \frac{F}{V}.$$

Here, M_{RNA} and $M_{protein}$ denote total cellular RNA and protein mass, respectively. F is the background-subtracted total fluorescence intensity of a cell and V is the volume of the cell.

Following are some quantifies used in the derivations below :

**Symbol**	**Meaning**
m_{rib}	Mass of ribosomal proteins in a single *E. coli* ribosome
m_{rRNA}	Mass of rRNA per ribosome
$m_{mChartreuse}$	Mass of a single mChartreuse molecule
N_{RspB}	Total number of RpsB molecules in an *E. coli* cell during steady state growth
N_{rib}	Total number of ribosomes in an *E. coli* cell during steady state growth
$N_{mChartreuse}$	Total number of mChartreuse molecules in an *E. coli* during steady state growth
M_{rib}	Mass of all ribosomal proteins in an *E. coli* cell
M_{RNA}	Mass of all RNAs in an *E. coli* cell
M_{rRNA}	Mass of all rRNAs in an *E. coli* cell
$M_{protein}$	Mass of all proteins in an *E. coli* cell
M_{cell}	Dry mass of an *E. coli* cell
R_{tr}	Transcription rate from a promoter in mass of mRNA generated per unit time
R_{tl}	Translation rate of a transcript in mass of peptide chain generated per unit time
R_d	Degradation rate of an mRNA/rRNA transcript
[g]	Gene number concentration in the cell
ϵ	Fluorescence output of a single mChartreuse molecule (even when in fusion with LacZ in the dual reporter, mChartreuse-LacZ)
C	DNA replication period in *E. coli*
V	Volume of an *E. coli* cell
λ	Growth rate of an *E. coli* cell

bioRxiv preprint doi: https://doi.org/10.64898/2026.09.17.752513; this version posted October 5, 2026. The copyright holder for this preprint (which was not certified by peer review) is the author/funder, who has granted bioRxiv a license to display the preprint in perpetuity. It is made available under a CC-BY 4.0 International license.

Relation between ribosome abundance and RNA-to-protein ratio (ρ)

Around 86% of RNA in exponentially growing *E. coli* cells is ribosomal RNA (rRNA) across multiple growth conditions (7):

$$M_{\mathrm{rRNA}} \approx 0.86\, M_{\mathrm{RNA}}. \tag{1}$$

In addition, ribosomal proteins and rRNA are produced in stoichiometric proportion (7, 8, 17):

$$M_{\mathrm{rRNA}} \propto M_{\mathrm{rib}}. \tag{2}$$

Combining equations (1) and (2) gives

$$M_{\mathrm{rib}} \propto M_{\mathrm{RNA}}. \tag{3}$$

In exponentially growing cells, cell density is approximately constant across multiple growth conditions (46), implying

$$V \propto M_{\mathrm{cell}}. \tag{4}$$

The protein fraction of biomass is also approximately constant over a range of growth conditions (38), so

$$M_{\mathrm{cell}} \propto M_{\mathrm{protein}}. \tag{5}$$

Combining equations (4) and (5) gives

$$V \propto M_{\mathrm{protein}}. \tag{6}$$

Equation (6) therefore implies

$$\frac{M_{\mathrm{rib}}}{M_{\mathrm{protein}}} \propto \frac{M_{\mathrm{rib}}}{V}. \tag{7}$$

Thus, ribosomal abundance $\left(\frac{M_{\mathrm{rib}}}{M_{\mathrm{protein}}}\right)$ is proportional to the ribosomal-protein concentration $\left(\frac{M_{\mathrm{rib}}}{V}\right)$ in an *E. coli* cell.

Using equation (3), we also obtain

$$\frac{M_{\mathrm{rib}}}{M_{\mathrm{protein}}} \propto \frac{M_{\mathrm{RNA}}}{M_{\mathrm{protein}}} \equiv \rho. \tag{8}$$

Hence, ribosomal abundance is proportional to the RNA-to-protein ratio ρ. As all the relations above hold across range of growth conditions, this relationship holds true for all the growth conditions studied here.

Relation between RpsB-mChartreuse fluorescence intensity (f) and RNA-to-protein ratio (ρ)

We assume that each mChartreuse (GFP) reporter contributes fluorescence ϵ. We call this the fluorescence output per reporter molecule in the main text. We also assume that the RpsB–mChartreuse fusion does not affect ribosome assembly, consistent with growth-phenotype data from earlier studies using RpsB–GFP fusions (28, 31). Because each ribosome contains one RpsB molecule, each ribosome is associated with one mChartreuse molecule.

The total cellular fluorescence is therefore

$$F = \epsilon N_{\mathrm{mChartreuse}}, \tag{9}$$

where $N_{\mathrm{mChartreuse}}$ is the number of mChartreuse molecules in the cell. We also have

$$N_{\mathrm{mChartreuse}} = N_{\mathrm{RpsB}} = N_{\mathrm{rib}} = \frac{M_{\mathrm{rib}}}{m_{\mathrm{rib}}}, \tag{10}$$

where we have used a single mChartreuse molecule per ribosome assumption.

bioRxiv preprint doi: https://doi.org/10.64898/2026.09.17.752513; this version posted October 5, 2026. The copyright holder for this preprint (which was not certified by peer review) is the author/funder, who has granted bioRxiv a license to display the preprint in perpetuity. It is made available under a CC-BY 4.0 International license.

Combining (9) and (10) with equation (7) gives

$$f = \frac{F}{V} = \epsilon \frac{N_{\text{rib}}}{V} = \frac{\epsilon}{m_{\text{rib}}} \frac{M_{\text{rib}}}{V} \propto \frac{M_{\text{rib}}}{M_{\text{protein}}}. \tag{11}$$

That is, the fluorescence intensity is expected to be proportional to the ribosomal abundance, under the assumption that the fluorescence output per unit reporter is constant. This along with equation (8) gives

$$f \propto \rho. \tag{12}$$

Hence, fluorescence intensity from the RpsB-mChartreuse translational fusion is expected to be directly proportional to the RNA-to-protein ratio.

Relation between fluorescence intensity (f) and RNA-to-protein ratio (ρ) in *rpsB::mChartreuse* and *rpsB::mChartreuse-lacZ*; also between [LacZ] and RNA-to-protein ratio (ρ) in *rpsB::mChartreuse-lacZ*

We assume again that each mChartreuse molecule contributes fluorescence ϵ.

In strains *rpsB::mChartreuse*, *rpsB::lacZ*, and *rpsB::mChartreuse-lacZ*, *mChartreuse*, *lacZ*, and *mChartreuse-lacZ* reporter constructs are downstream of the *rpsB* gene. However, both the reporter construct and the *rpsB* gene share the same promoter. Hence, both will have the same rate of transcription (R_{tr}). By construction mChartreuse in SJ2570 (*rpsB::mChartreuse*), LacZ in SJ2571 (*rpsB::lacZ*) and mChartreuse-LacZ in SJ2574 (*rpsB::mChartreuse-lacZ*) have the same ribosome binding site and hence the same translation rate (R_{tl}). RpsB, however, will have a different translation rate because it has a different ribosome binding site.

The steady-state concentration of RpsB, mChartreuse, LacZ and mChartreuse-LacZ protein molecules would be given by solving a system of coupled ODEs at steady state, describing the rate of change of mRNA concentration:

$$\frac{d[\text{mRNA}]}{dt} = R_{\text{tr}}[g] - (R_{\text{d}} + \lambda)[\text{mRNA}], \tag{13}$$

and rate of change of protein concentration

$$\frac{d[\text{Protein}]}{dt} = R_{\text{tl}}[\text{mRNA}] - \lambda[\text{Protein}], \tag{14}$$

where parameters are defined in the table above. These equations apply for RpsB, mChartreuse, LacZ and mChartreuse-LacZ reporter molecules, which share the same R_{tr}, R_{d}, but have different R_{tl}. For mRNA, the degradation rate (R_{d}) is much higher than the dilution rate due to the growth (λ), so that we can ignore the growth dilution of mRNA. We have also ignored the protein degradation rate, for it is negligible with respect to growth dilution in the conditions considered here.

Then solution at steady state gives the RpsB concentration ([RpsB]) as

$$[\text{RpsB}] = \frac{M_{\text{RpsB}}}{V} = \frac{R_{\text{tl}}^{\text{RpsB}}\ R_{\text{tr}}\ [g_{\text{RpsB}}]}{\lambda\ R_{\text{d}}}, \tag{15}$$

where $R_{\text{tl}}^{\text{RpsB}}$ is the translation rate for RpsB and $[g_{\text{RpsB}}]$ is the gene concentration of RpsB.

The concentration of mChartreuse ([mChartreuse]) in SJ2570 (*rpsB::mChartreuse*) is given by

$$[\text{mChartreuse}] = \frac{M_{\text{mChartreuse}}}{V} = \frac{R_{\text{tl}}^{\text{mChartreuse}}\ R_{\text{tr}}\ [g_{\text{mChartreuse}}]}{\lambda\ R_{\text{d}}}, \tag{16}$$

The solution should be analogous for [LacZ] and [mChartreuse-LacZ] :

bioRxiv preprint doi: https://doi.org/10.64898/2026.09.17.752513; this version posted October 5, 2026. The copyright holder for this preprint (which was not certified by peer review) is the author/funder, who has granted bioRxiv a license to display the preprint in perpetuity. It is made available under a CC-BY 4.0 International license.

$$[\text{LacZ}] = \frac{M_{\text{LacZ}}}{V} = \frac{R_{\text{tl}}^{\text{LacZ}}\ R_{\text{tr}}\ [g_{\text{LacZ}}]}{\lambda\ R_{\text{d}}}, \tag{17}$$

$$[\text{mChartreuse-LacZ}] = \frac{M_{\text{mChartreuse-LacZ}}}{V} = \frac{R_{\text{tl}}^{\text{mChartreuse-LacZ}}\ R_{\text{tr}}\ [g_{\text{mChartreuse-LacZ}}]}{\lambda\ R_{\text{d}}}, \tag{18}$$

Because mChartreuse and RpsB are in the same operon in SJ2570,

$$[g_{\text{RpsB}}] = [g_{\text{mChartreuse}}].$$

Similarly for LacZ in SJ2571 and mChatreuse-LacZ SJ2574, we have

$$[g_{\text{RpsB}}] = [g_{\text{LacZ}}],$$

$$[g_{\text{RpsB}}] = [g_{\text{mChartreuse-LacZ}}].$$

Even though mChartreuse and RpsB have different ribosome binding sites in SJ2570, we can assume that their translation rates are proportional to each other across growth conditions, so that

$$R_{\text{tl}}^{\text{mChartreuse}} \propto R_{\text{tl}}^{\text{RpsB}}.$$

Similarly for SJ2571 and SJ2574,

$$R_{\text{tl}}^{\text{LacZ}} \propto R_{\text{tl}}^{\text{RpsB}},$$

$$R_{\text{tl}}^{\text{mChartreuse-LacZ}} \propto R_{\text{tl}}^{\text{RpsB}}.$$

Then equations (15), (16), (17) and (18) imply

$$[\text{mChartreuse}] = \frac{M_{\text{mChartreuse}}}{V} \propto \frac{M_{\text{RpsB}}}{V}, \tag{19}$$

$$[\text{LacZ}] = \frac{M_{\text{LacZ}}}{V} \propto \frac{M_{\text{RpsB}}}{V}, \tag{20}$$

$$[\text{mChartreuse-LacZ}] = \frac{M_{\text{mChartreuse-LacZ}}}{V} \propto \frac{M_{\text{RpsB}}}{V}. \tag{21}$$

Then for the fluorescence intensity (f) from mChartreuse in SJ2570, we get

$$\begin{aligned} f = \frac{F}{V} &= \frac{\epsilon N_{\text{mChartreuse}}}{V} \\ &= \frac{\epsilon}{V}\frac{M_{\text{mChartreuse}}}{m_{\text{mChartreuse}}} \\ &\propto \frac{M_{\text{mChartreuse}}}{V}. \end{aligned} \tag{22}$$

A similar argument should follow for mChartreuse-LacZ in SJ2574.

Because there is a single RpsB molecule per ribosome,

bioRxiv preprint doi: https://doi.org/10.64898/2026.09.17.752513; this version posted October 5, 2026. The copyright holder for this preprint (which was not certified by peer review) is the author/funder, who has granted bioRxiv a license to display the preprint in perpetuity. It is made available under a CC-BY 4.0 International license.

$$\frac{M_{\text{RpsB}}}{V} = \frac{m_{\text{RpsB}}}{m_{\text{rib}}} \frac{M_{\text{rib}}}{V}.$$

This, along with equations (19), (20), (21), and (22), implies

$$f \propto \frac{M_{\text{rib}}}{V},$$

$$[\text{LacZ}] \propto \frac{M_{\text{rib}}}{V},$$

$$[\text{mChartreuse-LacZ}] \propto \frac{M_{\text{rib}}}{V}.$$

Hence fluorescence intensity and LacZ concentrations are proportional to ribosome concentration, which along with equations (7) and (8), give

$$f \propto \rho,$$

$$[\text{LacZ}] \propto \rho,$$

$$[\text{mChartreuse-LacZ}] \propto \rho.$$

Note here, we defined [LacZ] as mass concentration - but using equation (6) we get that LacZ mass concentration is proportional to LacZ mass as a fraction of total protein mass, as defined in the main text.

Relation between mChartreuse fluorescence intensity (f), [LacZ] and RNA-to-protein ratio (ρ) in ectopically expressed PrrnB*::mChartreuse*, PrrnE*::mChartreuse*, and PrrnB*::mChartreuse-lacZ* strains

Assume that each mature mChartreuse molecule contributes fluorescence ϵ.
The native *rrnB* operon and the ectopic *mChartreuse* reporter gene are controlled by the same *rrnB* promoter sequence. Therefore, their transcription rates per gene copy are assumed to be proportional:

$$R_{\text{tr}}^{\text{mChartreuse}} \propto R_{\text{tr}}^{\text{rrnB}}.$$

For the PrrnE::*mChartreuse* construct, the corresponding assumption is

$$R_{\text{tr}}^{\text{mChartreuse}} \propto R_{\text{tr}}^{\text{rrnE}}.$$

Following equation (13), the steady-state cellular concentration of rRNA transcribed from the native *rrnB* operon is

$$\frac{M_{\text{rRNA}}}{V} \propto \frac{R_{\text{tr}}^{\text{rrnB}}\,[g_{\text{rrnB}}]}{R_{\text{d}} + \lambda},$$

where $R_{\text{tr}}^{\text{rrnB}}$ is the transcription rate per native *rrnB* gene copy, $[g_{\text{rrnB}}]$ is the gene concentration of the native *rrnB* operon, R_{d} is the rRNA degradation rate and λ is the growth rate. The proportionality sign, instead of equality, is because rRNA expressed from other *rrn* operons also contribute to M_{rRNA}. Because rRNA is stable and only a small fraction of it is degraded in growth conditions of this study, R_{d} is assumed negligible with respect to λ. Then we get,

bioRxiv preprint doi: https://doi.org/10.64898/2026.09.17.752513; this version posted October 5, 2026. The copyright holder for this preprint (which was not certified by peer review) is the author/funder, who has granted bioRxiv a license to display the preprint in perpetuity. It is made available under a CC-BY 4.0 International license.

$$\frac{M_{\mathrm{rRNA}}}{V} \propto \frac{R_{\mathrm{tr}}^{\mathrm{rrnB}}\ [g_{\mathrm{rrnB}}]}{\lambda}. \tag{23}$$

Again following equations (13), (14) and short-half life of mRNA, the steady-state mChartreuse protein concentration is

$$\frac{M_{\mathrm{mChartreuse}}}{V} = \frac{R_{\mathrm{tl}}^{\mathrm{mChartreuse}}\ R_{\mathrm{tr}}^{\mathrm{mChartreuse}}\ [g_{\mathrm{mChartreuse}}]}{\lambda\ R_{\mathrm{d}}}. \tag{24}$$

The relationship between the native *rrnB* gene concentration and the ectopic reporter-gene concentration is written as (47)

$$[g_{\mathrm{mChartreuse}}] = e^{-xC\lambda}[g_{\mathrm{rrnB}}].$$

Similarly for rrnE reporter strain

$$[g_{\mathrm{mChartreuse}}] = e^{-xC\lambda}[g_{\mathrm{rrnE}}].$$

where C is the period DNA replication in *E. coli*, and x is the coordinate of the ectopic site, defined such that $x = 0$ is *oriC* and $x = 1$ is *ter*.

Transcriptional activity of the ectopic PrrnB reporter is expected to be approximately the same as transcription from the native rrnB operon, so that

$$R_{\mathrm{tr}}^{\mathrm{mChartreuse}} \approx R_{\mathrm{tr}}^{\mathrm{rrnB}}.$$

With the above relations and the assumption that the translation rate $R_{\mathrm{tl}}^{\mathrm{mChartreuse}}$ and degradation rate R_{d} do not change across the growth conditions, equation (24) gives

$$\frac{M_{\mathrm{mChartreuse}}}{V} \approx \frac{R_{\mathrm{tl}}^{\mathrm{mChartreuse}}\ R_{\mathrm{tr}}^{\mathrm{rrnB}}\ e^{-xC\lambda}\ [g_{\mathrm{rrnB}}]}{\lambda\ R_{\mathrm{d}}}$$

Then using equation (23) we have

$$\frac{M_{\mathrm{mChartreuse}}}{V} \approx \frac{R_{\mathrm{tl}}^{\mathrm{mChartreuse}} e^{-xC\lambda}}{R_{\mathrm{d}}} \frac{M_{\mathrm{rRNA}}}{V}. \tag{25}$$

In our case, $x = 0.64$ for SJ2560 (PrrnB::*mChartreuse*) and SJ2561 (PrrnE::*mChartreuse*) strains, which have the insertion near the *tolA* gene and $x = 0.75$ for SJ2574 (PrrnB::*mChartreuse-lacZ*) strain, which has the insertion is near the *appA* gene. We assume that $C = 40$ minutes $= 0.667$ hrs (4, 48) across all the nutrient-limited growth conditions. Thus, between the lowest ($\lambda = 0.25$/hr) and highest ($\lambda = 1.25$/hr) nutrient-limited growth rates in our study, the exponential factor $e^{-xC\lambda}$ leads to a 35-40% change. This change is smaller compared to the more than 400% change observed in the activity of PrrnB and PrrnE promoters (20, 32). During chloramphenicol inhibition, Si *et al.* (4) report a 4 times increase in C between $0\,\mu$M to $10\,\mu$M, however concomitantly there is a 4-fold decrease in growth rate (λ). This makes the exponential factor $e^{-xC\lambda}$ approximately constant over the chloramphenicol treatment range. Following these two arguments we can absorb the exponential factor into the proportionality. Then we get from equation (25):

$$\frac{M_{\mathrm{mChartreuse}}}{V} \propto \frac{R_{\mathrm{tl}}^{\mathrm{mChartreuse}}}{R_{\mathrm{d}}} \frac{M_{\mathrm{rRNA}}}{V},$$

and using equations (1) and (6),

bioRxiv preprint doi: https://doi.org/10.64898/2026.09.17.752513; this version posted October 5, 2026. The copyright holder for this preprint (which was not certified by peer review) is the author/funder, who has granted bioRxiv a license to display the preprint in perpetuity. It is made available under a CC-BY 4.0 International license.

$$\frac{M_{\text{mChartreuse}}}{V} \propto \frac{M_{\text{RNA}}}{M_{\text{protein}}}$$
$$\propto \rho.$$

Following the arguments of previous sections,

$$f = \frac{F}{V} \propto \frac{M_{\text{mChartreuse}}}{V},$$

and hence,

$$f \propto \rho.$$

This argument similarly applies to fluorescence intensity (f) and [mChartreuse-LacZ] in SJ2574 (PrrnB::*mChartreuse-lacZ*).

bioRxiv preprint doi: https://doi.org/10.64898/2026.09.17.752513; this version posted October 5, 2026. The copyright holder for this preprint (which was not certified by peer review) is the author/funder, who has granted bioRxiv a license to display the preprint in perpetuity. It is made available under a CC-BY 4.0 International license.

Supplementary figure 1

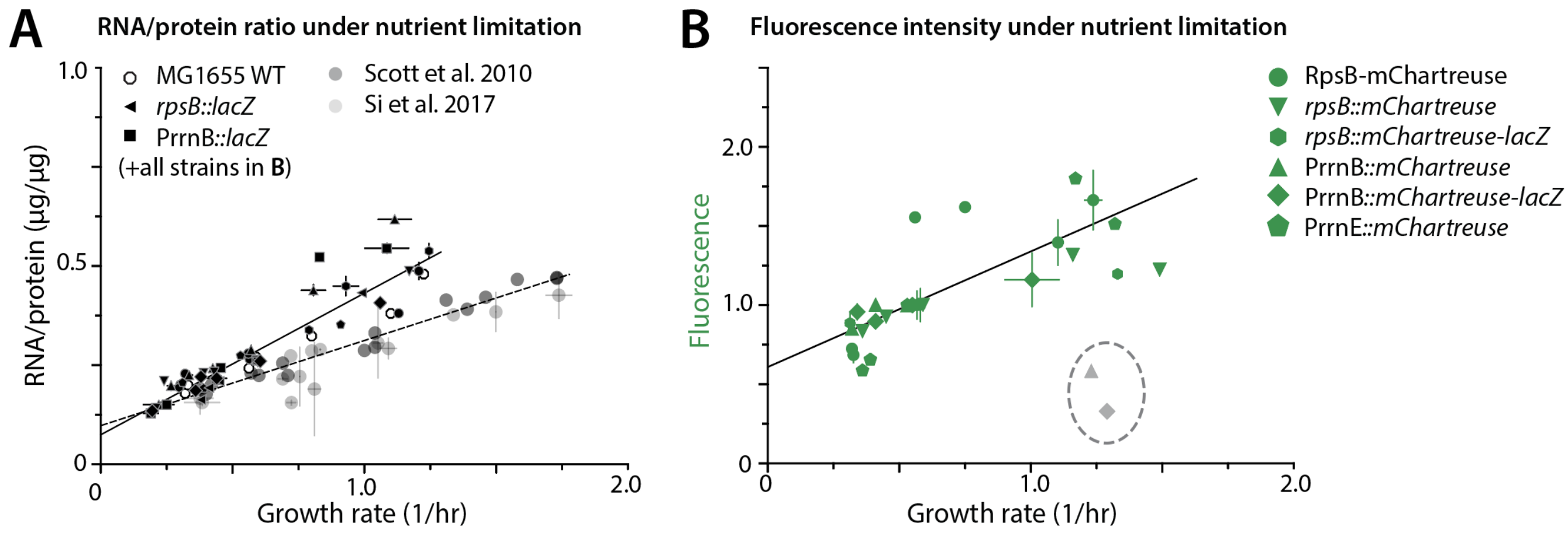

Figure S1 - RNA-to-protein mass ratio and Fluorescence intensity increase linearly with growth rate under nutrient limitation (**A**) RNA-to-protein mass ratio (RNA/protein) against growth rate under nutrient-limited growth, for wild-type MG1655, the *lacZ* reporter strains, and all mChartreuse reporter strains listed in **B**. Grey symbols are previously published measurements (3, 4). Solid line, fit to the strains of this study; dashed line, fit to the published data. (**B**) Fluorescence intensity against growth rate under the same conditions. Fluorescence is normalized to the value measured in MOPS glucose for each strain and hence it is unit less. Grey points are fluorescence data for PrrnB::*mChartreuse* and PrrnB::*mChartreuse-lacZ* in MOPS Rich Glycerol. For these growth conditions, the fluorescence values were much lower than other data points for the same strains. We are unable to explain this unexpected drop in fluorescence in MOPS Rich Glycerol. They were not included in the fit. Error bars are calculated as standard error of the mean between at least two replicates. Replicates information is given in Data S1.

Supplementary figure 2

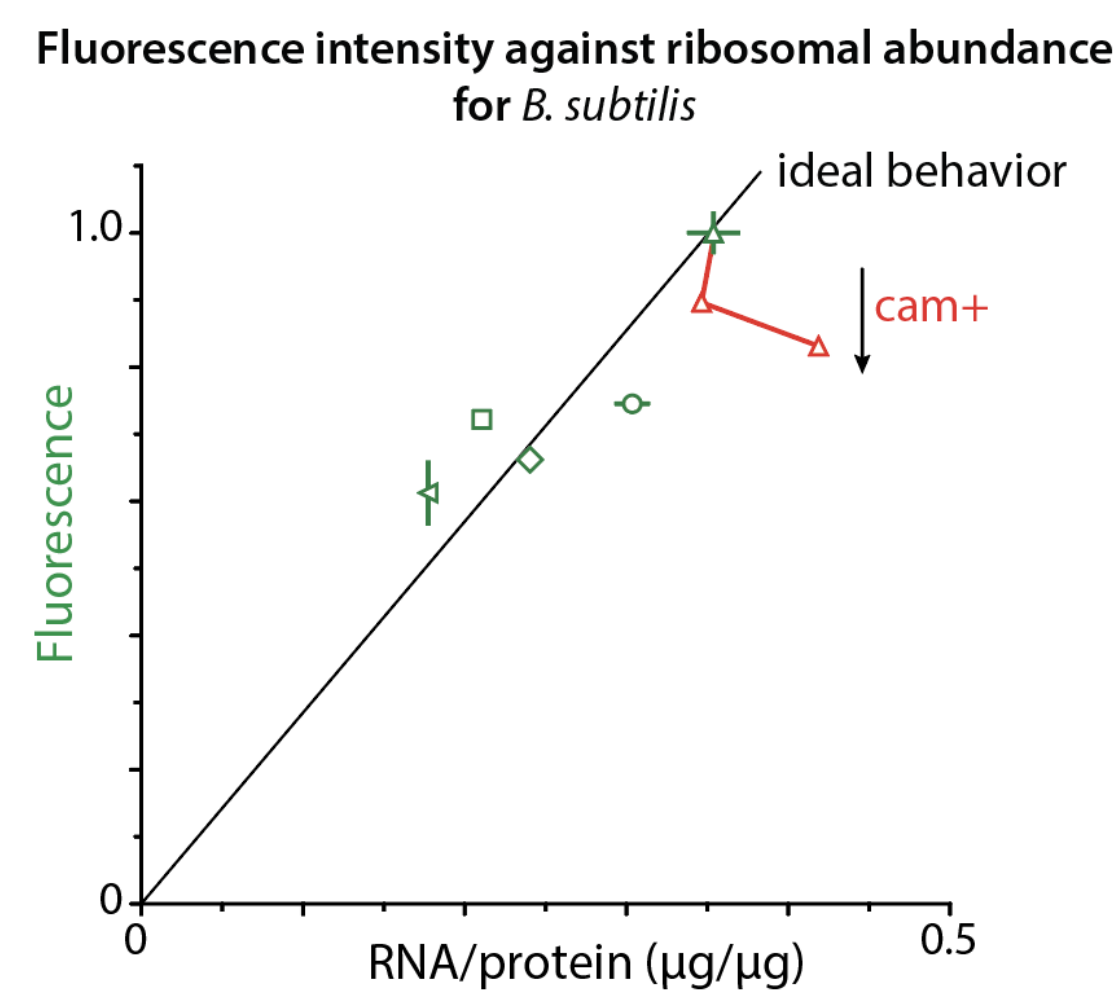

Figure S2 - Fluorescence intensity and RNA/protein relation for *B. subtilis* RpsB-mChartreuse. The growth media are given in Table 3. The ideal behaviour line is fit to the data forcing the intercept zero. Chloramphenicol was added to $S7_{50}$ Glucose with concentrations 4μM and 6μM. Error bars are calculated as standard error of the mean between at least two replicates. Replicates information is given in Data S1.

bioRxiv preprint doi: https://doi.org/10.64898/2026.09.17.752513; this version posted October 5, 2026. The copyright holder for this preprint (which was not certified by peer review) is the author/funder, who has granted bioRxiv a license to display the preprint in perpetuity. It is made available under a CC-BY 4.0 International license.

Data S1

Data generated in this study. For all the strains, data across different growth media and chloramphenicol (cam) concentrations are provided. In each growth condition, Average (Avg), Standard error of mean (SEM), and number of replicates (Replicates) are provided for Growth rates (1/hr), RNA/protein (μg/μg) values, Fluorescence intensities (normalized by MOPS Glucose fluorescence intensity), and [LacZ] (μg/μg) values.
